# Distinct Migration and Contact Dynamics of Resting and IL-2-Activated Human Natural Killer Cells

**DOI:** 10.3389/fimmu.2014.00080

**Published:** 2014-03-07

**Authors:** Per E. Olofsson, Elin Forslund, Bruno Vanherberghen, Ksenia Chechet, Oscar Mickelin, Alexander Rivera Ahlin, Tobias Everhorn, Björn Önfelt

**Affiliations:** ^1^Department of Applied Physics, Science for Life Laboratory, KTH Royal Institute of Technology, Stockholm, Sweden; ^2^Department of Microbiology, Tumor and Cell Biology, Karolinska Institute, Stockholm, Sweden

**Keywords:** natural killer cells, cell migration, single-cell, fluorescence imaging, microchip

## Abstract

Natural killer (NK) cells serve as one of the first lines of defense against viral infections and transformed cells. NK cell cytotoxicity is not dependent on antigen presentation by target cells, but is dependent on integration of activating and inhibitory signals triggered by receptor–ligand interactions formed at a tight intercellular contact between the NK and target cell, i.e., the immune synapse. We have studied the single-cell migration behavior and target-cell contact dynamics of resting and interleukin (IL)-2-activated human peripheral blood NK cells. Small populations of NK cells and target cells were confined in microwells and imaged by fluorescence microscopy for >8 h. Only the IL-2-activated population of NK cells showed efficient cytotoxicity against the human embryonic kidney 293T target cells. We found that although the average migration speeds were comparable, activated NK cells showed significantly more dynamic migration behavior, with more frequent transitions between periods of low and high motility. Resting NK cells formed fewer and weaker contacts with target cells, which manifested as shorter conjugation times and in many cases a complete lack of post-conjugation attachment to target cells. Activated NK cells were approximately twice as big as the resting cells, displayed a more migratory phenotype, and were more likely to employ “motile scanning” of the target-cell surface during conjugation. Taken together, our experiments quantify, at the single-cell level, how activation by IL-2 leads to altered NK cell cytotoxicity, migration behavior, and contact dynamics.

## Introduction

Natural killer (NK) cells are large granular lymphocytes capable of clearing both virus-infected and transformed cells. They have conventionally been classified as part of the innate immune system, but this picture is currently changing as studies have shown that NK cells display features of immunological memory normally ascribed to adaptive immunity (1). NK cell-mediated cytotoxicity is controlled by the integration of activating and inhibitory receptor signaling at the NK cell immune synapse (IS) formed between NK and target cell (2, 3). NK cells can also respond by producing cytokines, e.g., interferon-γ (IFN-γ) or tumor necrosis factor-α (TNF-α), and are known to be activated by cytokines like interleukin (IL)-2, IL-12, and IL-15.

Interleukin-2, initially called T-cell growth factor for its capacity to maintain *in vitro* cultures of primary T cells (4–6), has been widely used to augment the cytotoxic activity of NK cells *in vitro* (7). The immunostimulatory properties of IL-2 have been used in cancer treatment (8) where it has also been shown to selectively lead to NK cell expansion when given in relatively low doses over extended periods of time (9). It is poorly understood under what conditions NK cells can be stimulated by endogenous IL-2, even though cross-talk between NK cells and IL-2-producing T cells has been reported, linking the innate and adaptive immune systems (10–12).

Interleukin-2 shifts the gene and cell surface receptor expression of NK cells. Activating receptors, such as DNAM-1, NKp44, and KLRB1, are upregulated while inhibitory receptors, like KIR2DL2 and KIR3DL3, are downregulated after exposure to IL-2 (13, 14). The expression of adhesion molecules is also higher on IL-2-activated cells, consistent with the observation that they form stronger conjugates than resting NK cells (12, 15). Increased cell–cell adhesion has been directly coupled to cytotoxicity, partly explaining why IL-2-activated NK cells show higher cytotoxic potential than resting NK cells. IL-2 stimulation has also been observed to restore the formation of filamentous (F)-actin and cytotoxicity in NK cells from patients suffering from Wiskott–Aldrich syndrome (WAS) (16).

Although IL-2 activation generally enhances NK cells’ ability to lyse target cells, resting NK cells can also efficiently lyse some target-cell types, e.g., the leukemia cell line K562 (13). Bryceson et al. used resting NK cells in a redirected lysis assay to systematically decipher the role of individual activating receptors in combination with LFA-1 (that was triggered by expression of ICAM-1 on the P815 target cells). Engagement of CD16 led to cytotoxicity, whereas none of the receptors NKp46, NKG2D, 2B4, CD2, or DNAM-1 triggered a cytotoxic response. In IL-2-activated NK cells, individual engagement of these receptors was sufficient to trigger cytotoxicity. Interestingly, when resting NK cells were stimulated through combinations of these receptors, e.g., NKG2D and 2B4, or 2B4 and DNAM-1, cytotoxic responses could be triggered (13). Thus, resting NK cells are able to lyse target cells but require the right combination of activating signals, and, therefore, seem more tightly regulated than IL-2-activated NK cells.

An emerging theme at the border between technology and biology is the development of methods probing the dynamics of many individual cells in parallel. This can be achieved, for example, by using microchip-based tools trapping cells over extended periods of time (17–20). Such approaches have provided insights into NK cell heterogeneity in terms of cytokine production, killing behavior, and migration (21–23). We also recently reported significant heterogeneity among individual IL-2-activated NK cells in terms of migration and cytotoxicity and, here, compare this data with resting NK cells (21, 24). We report dramatic differences in morphology, contact dynamics, and target-cell killing, but less obvious differences in migration dynamics between resting and IL-2-activated cells.

## Materials and Methods

### Cells

Peripheral blood mononuclear cells were obtained from buffy coats of anonymous healthy donors and all experiments were performed in accordance with local ethics regulations. NK cells were isolated by negative selection according to manufacturer’s instructions (StemSep, StemCell Technologies, Grenoble, France; Miltenyi Biotec, Bergisch Gladbach, Germany) and cultured in IMDM or RPMI supplemented with 10% human serum, 50 U/ml penicillin-streptomycin, 1 × non-essential amino acids, 1 mM sodium pyruvate, and 50 μM β-mercaptoethanol (in some cultures only). Resting NK cells were used within 24–48 h of isolation. Activated NK cells were cultured in the same medium as above supplemented with 100 U/ml recombinant IL-2. Activated cells were used after 7–16 days. The purity of CD3^−^CD56^+^ cells was assessed by flow cytometry and was >95% for all experiments except one, for which CD3^−^CD56^+^ was >85%. For all isolations, the fraction of contaminating CD3^+^CD56^−^ T cells was <1%.

Human embryonic kidney (HEK) 293T (ATCC, Manassas, VA, USA) cells were used as target cells and were maintained in high-glucose RPMI-1640 supplemented with 10% FBS and 50 U/ml penicillin-streptomycin and for some experiments with additions of 1 × non-essential amino acids, 1 mM sodium pyruvate, and 50 μM β-mercaptoethanol.

### Cell labeling

Natural killer cells and HEK293T target cells were labeled for 10 min at 37°C in serum-free medium with 0.32–1 μM calcein red–orange and 1 μM calcein green (both Invitrogen), respectively. Cells were washed three times in serum-free medium prior to seeding in microwells.

### Microwell migration and cell–cell interaction imaging assay

The microchip-based imaging assay has been described in detail for IL-2-activated NK cells (21, 24). For resting NK cells, washed and sterilized microchips (vertical walls of 300 μm and base area 450 × 450 μm^2^) were rinsed with filtered PBS and coated with a 25 μg/ml fibronectin solution for 1 h at room temperature. Thereafter, the chip was mounted into a holder, rinsed with PBS, and covered with complete cell culture medium. Approximately 40,000 HEK 293T target cells were seeded onto the microwell chip and left to sediment and adhere to the glass bottom for 1.5–3 h at 37°C, 5% CO_2_. Afterward, approximately 20,000 NK cells were seeded onto the chip and left to sediment for 5 min. The upper layer of the medium was aspirated and replaced with new medium 10 times to remove unseeded NK cells, reducing the number of NK cells falling down into the wells during imaging. The average number of target and NK cells in each well was approximately 120 and 35, respectively. Fluorescence imaging was performed using a confocal microscope (LSM 510, Carl Zeiss, Oberkochen, Germany) with an open pinhole to maximize detected light. Up to four individual microwells in three separate experiments were imaged every 2 min for up to 12 h.

### Effects of differences in experimental protocols

Of note, the experimental setup differed slightly between resting and three out of four experiments of IL-2-activated NK cells. For all experiments in the resting condition and one in the IL-2-activated (*n* = 48), wells were coated with fibronectin and had slightly smaller base area (450 × 450 vs. 650 × 650 μm^2^). Fibronectin coating has been shown to facilitate migration of human NK cells through transwell systems (25) and maintain the survival of murine NK cells *in vitro* (26). Thus, any effects caused by fibronectin coating could be expected to lead to more migration of the resting NK cells and therefore decrease the overall differences in migration observed between the two conditions. Furthermore, NK cells close to the microwell walls could experience some restriction in their migration. Based on NK cell size, it is reasonable to assume that only NK cells within 15 μm from the walls would be affected by interactions with the walls. This area corresponds to 7 and 5% of the total area for the smaller and larger wells, respectively. Thus, any edge effects caused by the different well sizes can be expected to be small. β-mercaptoethanol was used in three out of four cultures of activated cells and although any effects are assumed to be negligible they cannot be ruled out.

### Cell tracking and analysis

Natural killer cells were tracked manually in the image analysis software packages Volocity (PerkinElmer, Waltham, MA, USA) and ImageJ. In total, 265 resting NK cells from four different donors (*n* = 113, 55, 69, 28) and 221 IL-2-activated NK cells from four different donors (*n* = 48, 50, 75, 48) were tracked in 8 h-long time-lapse movies. Each NK–target cell interaction was scored for duration (conjugation and attachment time) and outcome (killing/non-killing). Target-cell death was determined by examining both intracellular calcein fluorescence decrease as well as visible signs of death, like plasma membrane blebbing or cell swelling as previously described (24). Unless otherwise stated in figure legends, the data presented derive from all resting and activated NK cells.

### Migration analysis

Natural killer cell migration behavior was analyzed from individual cell trajectories similar to what has been described previously (20, 21). In short, cellular speed was calculated by comparing consecutive coordinates in the trajectories. Properties of each NK cell trajectory was quantified by calculating a local mean-square displacement (MSD) and migration coefficient using a sliding window of 25 time points centered around the time point to be calculated. Based on transient values of the migration coefficient and curvature of the MSD function, each NK cell trajectory was divided into different modes of migration [random movement, directed migration, and transient migration arrest periods (TMAPs)]. TMAPs are characterized by low motility that is typically confined to a small area. Directed migration is generally marked by higher and directionally persistent migration. Random movement is defined as neither TMAP nor directed migration, i.e., motion that appears stochastic and consistent with random-walk.

### Statistical analysis

The non-parametric two-sided Mann–Whitney *U*-test was used to evaluate statistical significance for all data except for that presented in Figure S6 in Supplementary Material, where the Wilcoxon signed-rank test was used. *p*-values < 0.05 were considered statistically significant. Data shown are mean ± standard deviation unless otherwise stated.

## Results

### Activated NK cells exhibit more dynamic migration, which is related to modes of migration

**Figure 1:**
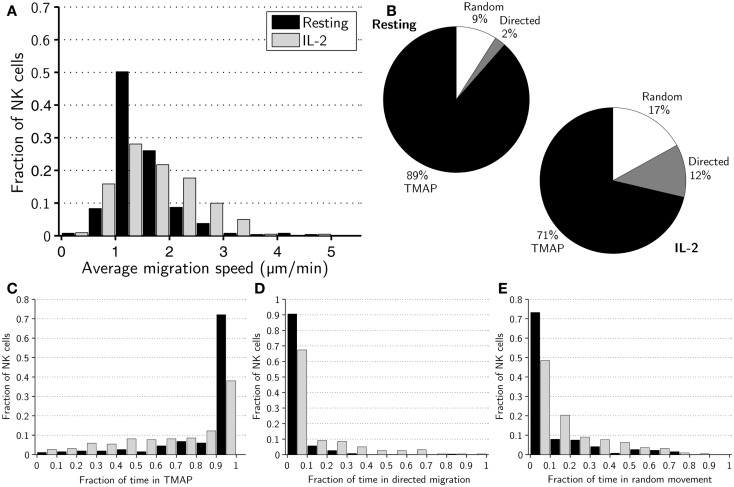
**Migration dynamics of resting and activated NK cells**. **(A)** Distributions of average migration speeds for resting (black bars) and activated (gray bars) NK cells. **(B)** Pie charts showing mean fraction of time spent in TMAPs (black), directed migration (gray), and random movement (white) for resting (left chart) and activated (right chart) NK cells. **(C)** Distributions of fraction of time spent in TMAPs for resting (black bars) and activated (gray bars) NK cells. **(D)** Distributions of fraction of time spent in directed migration for resting (black bars) and activated (gray bars) NK cells. **(E)** Distributions of fraction of time spent in random movement for resting (black bars) and activated (gray bars) NK cells.

The average mean migration speed of the resting NK cells over the 8-h assay was ±0.6 μm/min while it was ±0.7 μm/min for activated NK cells (*p* < 0.005). A histogram of mean migration speeds revealed substantial differences in the mean speeds of individual cells within subsets and that fast-migrating NK cells were more common in the activated subset (Figure 1A).

We then set out to investigate if there were any detectable differences in the migration behavior of activated and resting NK cells. To this end, we applied a previously developed method to sub-divide cell trajectories into three distinct modes of migration, i.e., TMAPs, directed migration or random movement (21). Overall, a majority of NK cells spent considerable time in TMAPs with the average fractions of time 89% for resting and 71% for activated NK cells (Figure 1B). Strikingly, approximately 72% of resting NK cells spent between 90 and 100% of the assay in TMAPs, compared to 38% for the activated NK cells (Figure 1C). Thus, a large fraction of the resting cells displayed low motility while activated NK cells were significantly more motile (*p* < 0.005).

Examining directionally persistent migration showed that all resting NK cells (with the exception of one cell) spent little time in directed migration (<40% of the time), while a few activated NK cells spent >40% of the assay in directed migration (Figure 1D). The average times spent in directed migration were 2% for resting NK cells and 12% for activated cells (Figure 1B). Thus, in this assay resting NK cells almost completely lacked directionally persistent migration. The rest of the time, on average 9% for resting NK cells and 17% for activated NK cells, was spent in random movement (Figures 1B,E). Analysis showed that differences between resting and activated cells in the fractions of time spent in different modes of migration were statistically significant (*p* < 0.005).

Next we compared the mean migration speeds of cells in different modes of migration and, as expected, the average mean speeds in TMAPs were considerably lower, ±0.8 μm/min for resting and ±0.8 μm/min for IL-2-activated NK cells (*p* < 0.05) compared to other modes of migration. In directed migration, the average mean speeds were ±1.1 and ±0.7 μm/min for resting and activated NK cells, respectively (*p* < 0.005). The random movement periods had average mean speeds of ±1.1 μm/min for resting and ±1.0 μm/min for activated NK cells (n.s; *p* = 0.09, Figure S1 in Supplementary Material). Thus, resting NK cells had higher average mean speeds in both directed migration and random movement and, yet, had a lower overall average mean speed.

Taken together, the observed shift in the distribution of migration modes (Figure 1B) for resting and activated NK cells shows that IL-2 gives the NK cells a more migratory phenotype. This difference was reflected in a slight skewing of the distribution toward higher mean speeds for activated cells but even more pronounced when looking at transient migration behavior.

### Activated NK cells more frequently alternate between different modes of migration

**Figure 2:**
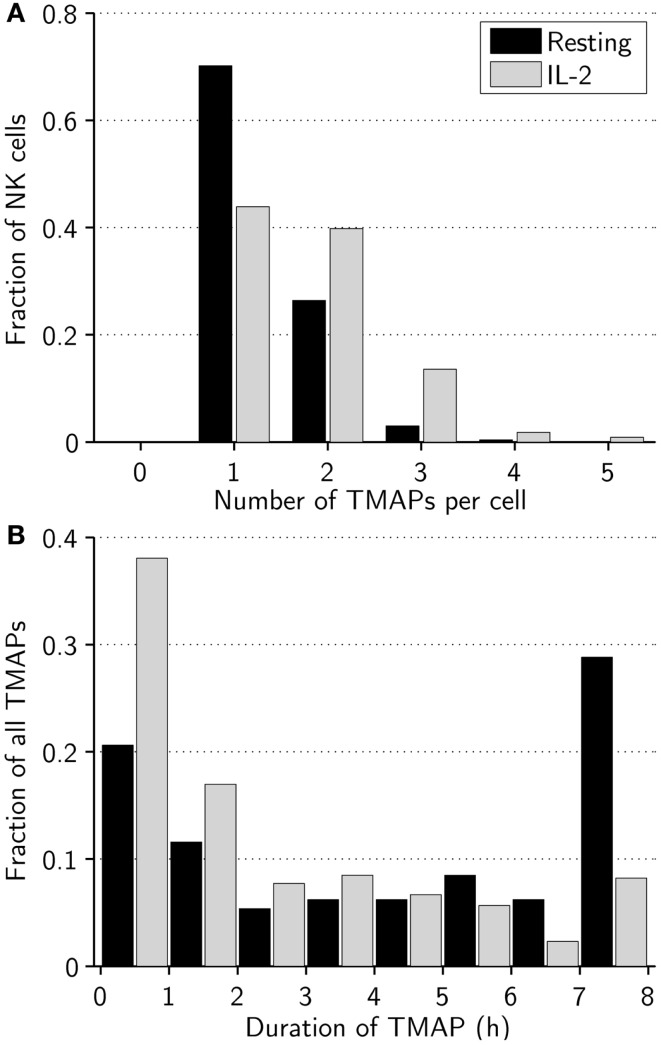
**Activated NK cells alter between different modes of migration more frequently than resting NK cells**. **(A)** Distribution of number of TMAPs recorded for individual resting (black bars) and activated (gray bars) NK cells. The number of TMAPs detected was 354 for resting and 389 for activated NK cells. **(B)** Distribution of duration of individual TMAPs for resting (black bars) and activated (gray bars) NK cells.

Next, we investigated whether other characteristic differences in migration modes existed between resting and activated NK cells. Overall, resting NK cells switched between different modes of migration on average 1.1 times during the assay, compared to 2.8 times for activated NK cells. As an example, we studied the number and duration of TMAPs for individual cells in the experiments (Figure 2). All NK cells studied had at least one TMAP, which was also the most frequent number of TMAPs in both subsets with decreasing frequency up to five TMAPs, which was the maximum observed. Looking at average values, resting NK cells made fewer (1.3 vs. 1.8, *p* < 0.005), but longer (272 vs. 169 min, *p* < 0.005) TMAPs compared to activated NK cells (Figures 2A,B). Additionally, approximately 30% of the TMAPs from the resting NK cell subset, threefold more than activated NK cells, lasted almost the entire assay indicating low to no motility. In contrast, activated NK cells showed approximately 30% more TMAPs that lasted less than 2 h compared to resting NK cells. This shows that while both cell populations exhibited stop-and-go behavior, it was more characteristic of activated NK cells.

### Striking differences in contact formation between resting and activated NK cells

**Figure 3:**
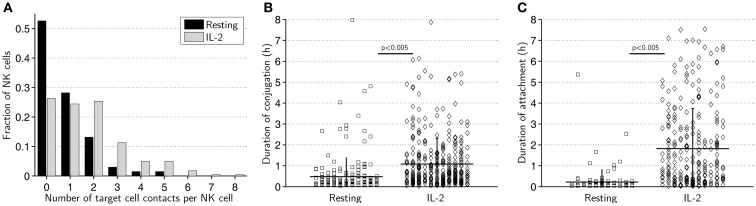
**Resting NK cells form fewer and shorter contacts compared to IL-2-activated NK cells**. **(A)** Distribution of number of contacts formed by resting (black bars) and activated (gray bars) NK cells. The total number of contacts recorded was 205 for resting and 379 for activated NK cells. **(B)** Duration of conjugation periods for resting (squares, left) and activated (diamonds, right) NK cells. **(C)** Duration of attachment periods for resting (squares, left) and activated (diamonds, right) NK cells.

Next, we compared the ability of activated and resting cells to form contacts with target cells. Activated NK cells were found to form up to eight contacts during the assay while the maximum was five for resting NK cells (Figure 3A). Strikingly, on average, activated NK cells formed more than twice as many contacts as resting NK cells (1.7 vs. 0.8, *p* < 0.005).

We next scored the duration of conjugation, where the NK cell assumes a rounded morphology with membrane flattening at the site of intercellular contact, and attachment, where the NK cell remains attached to the target cell but visibly seeks to migrate away (24). Both conjugation (Figure 3B) and attachment (Figure 3C) phases were significantly longer for activated NK cells than resting NK cells. On average, activated NK cells remained conjugated to target cells twice as long (65 vs. 29 min, *p* < 0.005) and attached nearly eight times as long (110 vs. 14 min, *p* < 0.005) as resting NK cells. A considerably larger fraction of resting NK cells completely lacked an attachment phase compared to activated NK cells (44 vs. 27%). These data imply that resting NK cells do not form as “strong” contacts as activated NK cells and are less likely to remain attached to target cells during termination of cell–cell contact.

### Donor-to-donor variations can partly explain the observed differences in migration – but not contact dynamics

Cells from distinct donors were used in the majority of experiments and to assess if any of the observed differences in migration and contact dynamics could be related to donor-to-donor differences rather than IL-2 activation, data for each donor was studied separately (Figure S3 in Supplementary Material). Statistical analysis revealed significant differences in terms of migration dynamics within resting and activated conditions and also overlapping distributions of mean values for the two experimental conditions. In contrast, separate analysis of contact and attachment times showed clearly that although some variations were detected within conditions, the mean values formed non-overlapping groups (Figure S3 in Supplementary Material). Thus, this suggests that donor-related effects cannot be ignored for the migration parameters measured. In terms of contact dynamics, however, IL-2-activation is clearly the dominant effect.

For one set of experiments, cells isolated from one donor was first studied under resting conditions and then again after 7 days in IL-2 culture (*n*_resting_ = 55 and *n*_activated_ = 48 in resting and activated conditions, respectively; Figure S4 in Supplementary Material). These data followed the same trend as the pooled data with smaller differences in migration properties (Figures S4A–C in Supplementary Material) but dramatic differences in contact times (Figures S4D,E in Supplementary Material).

### Motile scanning and speed in contact

**Figure 4:**
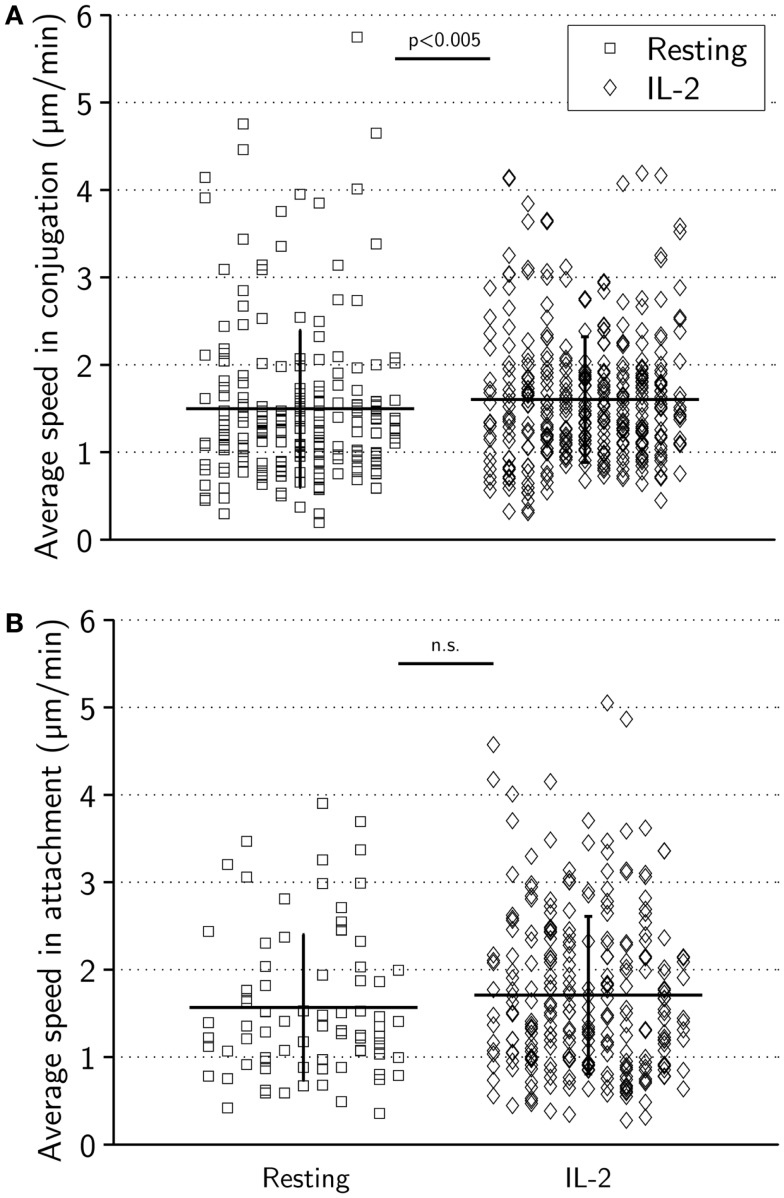
**Average speeds during target-cell contact are similar for resting and activated NK cells**. **(A)** Average speed in conjugation for resting (left) and activated (right) NK cells. Mean ± SD was 1.5 ± 0.9 μm/min for resting and 1.6 ± 0.7 μm/min for activated cells. **(B)** Average speed in attachment for resting (squares, left) and activated (diamonds, right) NK cells. Mean ± SD was 1.6 ± 0.8 μm/min for resting and 1.7 ± 0.9 μm/min for activated cells.

When in conjugation with target cells, resting and activated NK cells had comparable average mean speeds (±0.9 μm/min for resting NK cells and ±0.7 μm/min for activated NK cells, *p* < 0.005) (Figure 4A). The average speed in attachment was slightly higher (±0.8 μm/min for resting NK cells and ±0.9 μm/min for activated NK cells) consistent with a more migratory morphology (Figure 4B). During attachment periods, in particular for activated NK cells, it was occasionally observed that NK cells dragged target cells along after termination of the conjugation phase (data not shown). While the difference in mean attachment speed was not statistically significant (*p* = 0.23), the difference in mean conjugation speed was *p* < 0.005. This could be explained by differences in the distribution of measured speeds, and indeed, some NK cells were observed to move at a considerable speed while in conjugation with target cells.

**Figure 5:**
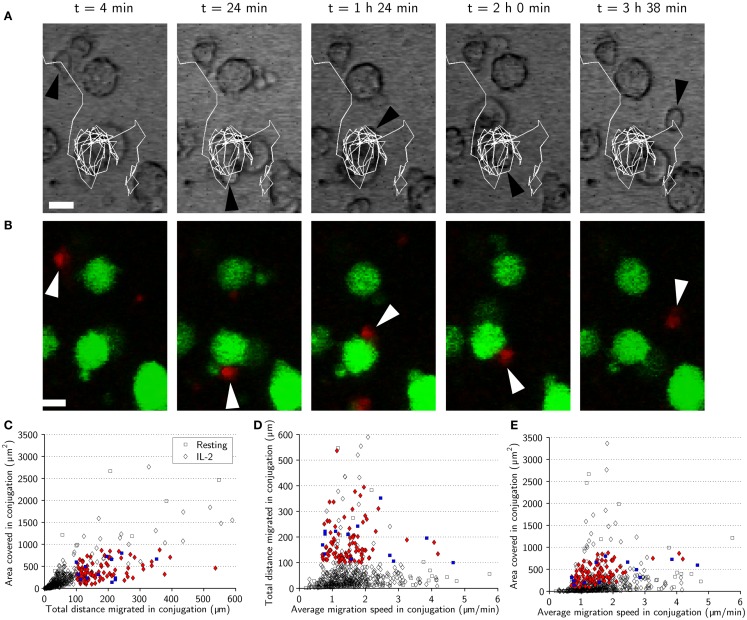
**Natural killer cell motile scanning of the target-cell surface**. **(A)** Time-lapse sequence showing an IL-2-activated NK cell (black arrowhead) scanning the surface of a target cell. The images are overlaid with a trace of the NK cell trajectory (white) showing that the NK cell moves several laps around the target cell before detaching. Scale bars indicate 10 μm. **(B)** Fluorescence images corresponding to **(A)** showing the fluorescently labeled NK cell (red, white arrowhead) and target cells (green). Both transmitted-light and fluorescence images have been resampled. **(C)** Total distance migrated in conjugation plotted vs. area covered in conjugation for resting (squares) and activated (diamonds) NK cells indicating the conjugation periods fulfilling the three criteria (blue squares and red diamonds for resting and activated NK cells, respectively). **(D)** Average migration speed in conjugation plotted vs. total distance migrated in conjugation for resting (squares) and IL-2-activated (diamonds) indicating motile scanning behavior (colored symbols). **(E)** Average migration speed in conjugation plotted vs. area covered in conjugation indicating motile scanning behavior (colored symbols).

During tracking we had observed that some NK cells remained highly motile, moving across the surface of target cells during conjugation (Figures 5A,B). In an attempt to investigate such “motile scanning” further, we identified three criteria to isolate subsets of conjugates consistent with this behavior. Those criteria were: (1) The total distance moved while in conjugation should be >100 μm. (2) The area covered by the NK cell during the conjugation period should be <900 μm^2^, corresponding to a rectangle with sides approximately similar to the diameter of up to three target cells (30 μm) and determined by the max and min values for *x* and *y* coordinates during conjugation. (3) The distance from the starting point of the conjugate to the endpoint should be <30 μm. The conjugation periods identified by these three criteria can be seen in a plot of area covered vs. total distance (Figure 5C). Among the colored markers used to denote conjugates exhibiting motile scanning also some unfilled symbols can be seen that were excluded based on the displacement criterion. The conjugates isolated by these criteria were not associated with increased speeds as shown in plots of total distance or area covered vs. mean speeds (Figures 5D,E). These criteria were satisfied by 25% of conjugates from the activated subset and 8% of the conjugates from the resting subset, suggesting that motile scanning is more common among activated NK cells.

### Activated NK cells have migratory morphology and spread across the target cell while resting cells remain round during synapse formation

**Figure 6:**
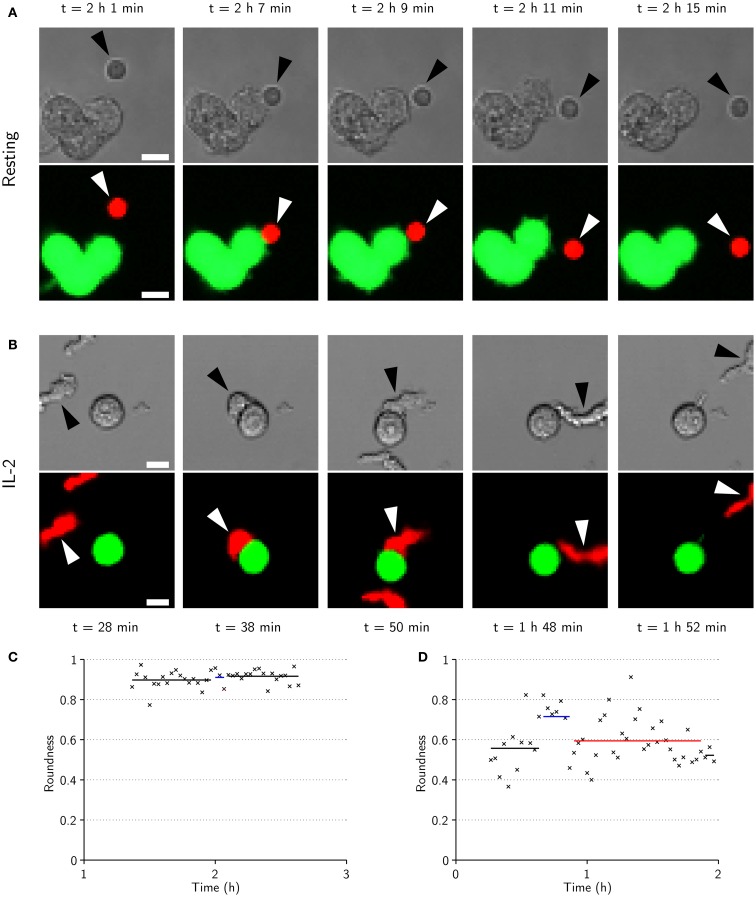
**IL-2-activated NK cells are larger, have elongated shapes during migration, and spread across the target cell during conjugation**. **(A)** Time-lapse sequence showing transmitted-light (top) and fluorescence (bottom) images of a resting NK cell (arrowheads) approaching (*t* = 2 h 1 min) a cluster of three target cells (green in fluorescence images). The NK cell is briefly conjugated to the target cell (*t* = 2 h 7 min), before it appears to end its commitment to the target and enter an attachment phase (*t* = 2 h 9 min) that ends as the NK cell detaches (*t* = 2 h 11 min) and resumes free migration (*t* = 2 h 15 min). Scale bars indicate 10 μm. **(B)** Same as in **(A)** but for an activated NK cell. The NK cell approaches a target cell (*t* = 28 min) and forms a conjugate (*t* = 38 min) that ends as the NK cell assumes a migratory morphology (*t* = 50 min). The NK cell remains attached to the target cell for almost an hour before it detaches (*t* = 1 h 48 min) and migrates away from the target cell (*t* = 1 h 52 min). **(C)** NK cell roundness vs. time for the sequence shown in **(A)**. **(D)** NK cell roundness vs. time for sequence shown in **(B)**. Shown are also mean roundness in conjugation (blue lines), attachment (red lines), and free migration (black lines).

Morphologically, resting cells appeared to be relatively small and round when migrating freely, and to maintain their shape upon target-cell contact (Figure 6A). By contrast, activated NK cells generally had more elongated and irregular shapes which, upon encounter with target cells, became more rounded with significant spreading across the target-cell surface, creating an intercellular contact that was flat and relatively large (Figure 6B). These observations are consistent with recent imaging studies of murine NK cells (12). Measurements of roundness and area of isolated resting (*n* = 106) and activated (*n* = 68) NK cells confirmed that resting NK cells generally were rounder and smaller (Figure S2 in Supplementary Material). Roundness and area were also measured for time sequences involving phases of free migration and synapse formation for the cells displayed in Figures 6A,B (Figures 6C,D) and a randomly selected subset of NK cells (*n* = 4 for each condition, Figure S5 in Supplementary Material). Transitions between different phases were reflected in changes in roundness and area for IL-2-activated but not resting NK cells. Thus, under the experimental conditions used here, freely migrating activated NK cells had an elongated, irregular shape that was altered to a rounded shape upon target-cell contact. In contrast, resting NK cells were smaller and appeared to maintain a similar morphology throughout the different phases of migration and target-cell contact.

### Marked difference in cytotoxicity of resting and activated NK cells toward HEK293T cells

Finally, single-cell cytolytic activity was compared for resting and activated NK cells. Both resting and activated NK cells displayed cytolytic activity against HEK 293T cells by standard chromium release, although resting NK cell lytic ability was reduced [(24) and data not shown]. A detailed analysis of single-cell level cytotoxicity of activated NK cells has been presented elsewhere (24). During the 8-h analysis, here, activated NK cells killed a total of 240 target cells, an average of 1.1 kills/NK cell. In sharp contrast, only two cytolytic events resulted from the 205 contacts made by the 265 resting NK cells analyzed. Thus, in our assay, resting NK cells showed a significantly lower ability to kill HEK293T cells than activated cells.

Recently, it was suggested by mathematical modeling (27), and later measured experimentally (28), that NK cells become more efficient killers after initial contact with target cells. In light of that, we analyzed the time to lytic hit, i.e., the time from initiation of conjugate to delivery of the lytic hit (24), for a subset of activated NK cells that performed more than one kill in direct sequence. A majority (59%) of NK cells delivered the lytic hit faster for the second conjugate compared to the first but statistical analysis could not confirm that this was significant (*n* = 54, *p* = 0.19).

## Discussion

Here, we have compared resting and IL-2-activated NK cells using a microchip-based method with single-cell resolution for resolving migration and NK–target-cell contact dynamics over extended periods of time. Although the difference in average migration speed was small between resting and activated NK cells, a more detailed analysis revealed that resting NK cells were less likely than activated cells to transiently switch between different modes of migration. Overall, activated NK cells were more dynamic with a broader range of morphologies consistent with alterations between migration, stopping, and immune synapse formation. These differences in dynamics are in line with previous imaging studies of NK cells *in vitro* with and without IL-2 activation (12), *in situ* under steady state and inflammatory conditions (21) and during tumor surveillance (29). Thus, it appears that activated NK cells are more dynamic independently of their means of stimulation.

Resting NK cells generally formed shorter contacts and, in contrast to activated cells, rapidly terminated conjugation without attaching to the target cell for prolonged periods of time. This is consistent with previous reports stating that IL-2 leads to upregulation of adhesion molecules and stronger conjugate formation (12, 15). Interestingly, it was recently shown that NK cell education/licensing also leads to increased formation of stable conjugates, emphasizing its importance for efficient cytotoxicity (30). However, the effect observed was not due to a general upregulation of the integrin LFA-1 in educated NK cells, but due to increased inside-out signaling triggered by activating receptors leading to increased expression of high-affinity LFA-1.

Activated NK cells more frequently formed dynamic contacts where the NK cell appeared to scan the surface of the target cell. This motile scanning could be related to “kinapses” described for T cells, a process believed to optimize the search for ligands on the target cell (31). At the same time, it is known that NK cells receiving sufficient activation signals will stop and spread out (32). Thus, it may seem paradoxical that motile scanning was more frequently observed for IL-2-activated NK cells, where conjugation and killing was much more efficient. However, it is known that some integrin interactions can promote migration, e.g., LFA-1/ICAM-1 (32). So considering that adhesion molecules are upregulated upon interleukin-2 stimulation is conceivable that motile scanning could indeed be more common among activated NK cells. More thorough studies are needed to better characterize motile scanning and to resolve its significance. It is for example possible that motile NK cells can integrate the signals generated by encountering spatially separated ligands, possibly expressed on separate cells. This is supported by data recently reported by us where a microwell array for ultrasonic manipulation was used to form clusters of single NK cells and varying numbers of target cells (23). Interestingly, NK cells surrounded by two or more target cells were more likely to kill compared to those in contact with only one target cell. Integration of signals from several targets could also be linked to “burst kinetics,” where NK cells were observed to kill faster after initial contact with target cells (28). In our data, there was a tendency of the second and third killing events committed by individual NK cells being faster than the first, but the effect could not be verified statistically. Of note is that we used a different cell system and that target cells were more spread out in the assay leading to subsequent killing events that could be separated both in space and in time, i.e., some NK cells had to detach from the first target and migrate to the next before killing could take place. Thus, simultaneous contact with several target cells was not the case for all NK cells and killing events in our assay.

The data presented here demonstrate that there are significant differences in migration and contact dynamics between resting and IL-2-activated NK cells. At the same time, there were also significant overlaps between the two populations reflecting inherent heterogeneity among peripheral blood NK cells and a varying response to activation. Importantly, differences in migration dynamics may be overlooked if analysis of transient behavior is not performed. The type of analysis performed here is only possible by studying the migration and contact history of all individual cells in a population over extended periods of time. Although this arguably could be achieved by conventional wide-field imaging, it is greatly facilitated by microchip-based approaches like the one used here. While there are several reports of heterogeneity of NK cells in terms of receptor expression and how that is related to activation, maturation or level of education, little is currently known about how this is reflected in migration dynamics, conjugate formation, and killing at the single-cell level (33). We foresee that methods allowing studies of single cells over time will become more widespread and help bridge the existing gaps in understanding of the behavior of individual cells and the function of cell populations.

## Author Contributions

Per E. Olofsson designed experiments, carried out experiments, analyzed data, and wrote the manuscript. Elin Forslund designed experiments, carried out experiments, and contributed to writing the manuscript. Bruno Vanherberghen designed experiments, carried out experiments, analyzed data, and contributed to writing the manuscript. Ksenia Chechet, Oscar Mickelin, Alexander Rivera Ahlin, and Tobias Everhorn analyzed data. Björn Önfelt designed experiments and wrote the manuscript.

## Conflict of Interest Statement

The authors declare that the research was conducted in the absence of any commercial or financial relationships that could be construed as a potential conflict of interest.

## Supplementary Material

The Supplementary Material for this article can be found online at http://www.frontiersin.org/Journal/10.3389/fimmu.2014.00080/abstract
