# Erratum: Distinct Migration and Contact Dynamics of Resting and IL-2-Activated Human Natural Killer Cells

**DOI:** 10.3389/fimmu.2014.00451

**Published:** 2014-09-22

**Authors:** Frontiers Production Office

**Affiliations:** ^1^Frontiers Production Office, Frontiers, Switzerland

**Keywords:** natural killer cells, cell migration, single-cell, fluorescence imaging, microchip

## Reason for Erratum

In the Section “Results” of the article, several numbers in the text were misinterpreted due to a typesetting error. This mistake does not change the scientific conclusions of the article in any way. The publisher apologizes for this error and the correct versions of the relevant paragraphs appear below.

The corrected values are in bold.

## Results

### Activated NK cells exhibit more dynamic migration, which is related to modes of migration

The average mean migration speed of the resting NK cells over the 8-h assay was **1.6 ± 0.6** μm/min, while it was **1.7 ± 0.7** μm/min for activated NK cells (*p* < 0.005). A histogram of mean migration speeds revealed substantial differences in the mean speeds of individual cells within subsets and that fast-migrating NK cells were more common in the activated subset (Figure 1A).

We then set out to investigate if there were any detectable differences in the migration behavior of activated and resting NK cells. To this end, we applied a previously developed method to sub-divide cell trajectories into three distinct modes of migration, i.e., TMAPs, directed migration, or random movement (21). Overall, a majority of NK cells spent considerable time in TMAPs with the average fractions of time 89% for resting and 71% for activated NK cells (Figure 1B). Strikingly, approximately 72% of resting NK cells spent between 90 and 100% of the assay in TMAPs compared to 38% for the activated NK cells (Figure 1C). Thus, a large fraction of the resting cells displayed low motility while activated NK cells were significantly more motile (*p* < 0.005).

Examining directionally persistent migration showed that all resting NK cells (with the exception of one cell) spent little time in directed migration (<40% of the time), while a few activated NK cells spent >40% of the assay in directed migration (Figure 1D). The average times spent in directed migration were 2% for resting NK cells and 12% for activated cells (Figure 1B). Thus, in this assay, resting NK cells almost completely lacked directionally persistent migration. The rest of the time, on average, 9% for resting NK cells and 17% for activated NK cells, was spent in random movement (Figures 1B,E). Analysis showed that differences between resting and activated cells in the fractions of time spent in different modes of migration were statistically significant (*p* < 0.005).

Next, we compared the mean migration speeds of cells in different modes of migration and, as expected, the average mean speeds in TMAPs were considerably lower, **1.7 ± 0.8** μm/min for resting and **1.7 ± 0.8** μm/min for IL-2-activated NK cells (*p* < 0.05) compared to other modes of migration. In directed migration, the average mean speeds were **3.0 ± 1.1** and **2.5 ± 0.7** μm/min for resting and activated NK cells, respectively (*p* < 0.005). The random movement periods had average mean speeds of **2.6 ± 1.1** μm/min for resting and **2.4 ± 1.0** μm/min for activated NK cells (n.s; *p* = 0.09, Figure S1 in Supplementary Material). Thus, resting NK cells had higher average mean speeds in both directed migration and random movement and, yet, had a lower overall average mean speed.

Taken together, the observed shift in the distribution of migration modes (Figure 1B) for resting and activated NK cells shows that IL-2 gives the NK cells a more migratory phenotype. This difference was reflected in a slight skewing of the distribution toward higher mean speeds for activated cells but even more pronounced when looking at transient migration behavior.

### Motile scanning and speed in contact

When in conjugation with target cells, resting and activated NK cells had comparable average mean speeds (**1.5 ± 0.9** μm/min for resting NK cells and **1.6 ± 0.7** μm/min for activated NK cells, *p* < 0.005) (Figure 4A). The average speed in attachment was slightly higher (**1.6 ± 0.8** μm/min for resting NK cells and **1.7 ± 0.9** μm/min for activated NK cells) consistent with a more migratory morphology (Figure 4B). During attachment periods, in particular for activated NK cells, it was occasionally observed that NK cells dragged target cells along after termination of the conjugation phase (data not shown). While the difference in mean attachment speed was not statistically significant (*p* = 0.23), the difference in mean conjugation speed was *p* < 0.005. This could be explained by differences in the distribution of measured speeds, and indeed, some NK cells were observed to move at a considerable speed while in conjugation with target cells.

## References

[21] Khorshidi MA Vanherberghen B Kowalewski JM Garrod KR Lindstrom S Andersson-Svahn H Analysis of transient migration behavior of natural killer cells imaged *in situ* and *in vitro*. Integr Biol (Camb) (2011) 3:770–8 10.1039/c1ib00007a 21687858

