# Supplementary material for: Distinct Migration and Contact Dynamics of Resting and IL-2-Activated Human Natural Killer Cells
Source: Front Immunol. 2014 Mar 7;5:80. doi: 10.3389/fimmu.2014.00080 (PMC3945532; doi:10.3389/fimmu.2014.00080)
Supplement: Supplementary file 1 [file Data_Sheet1.PDF]

- Supplementary material -

Distinct migration and contact dynamics of resting and IL-2-activated human natural killer cells

Olofsson et al.

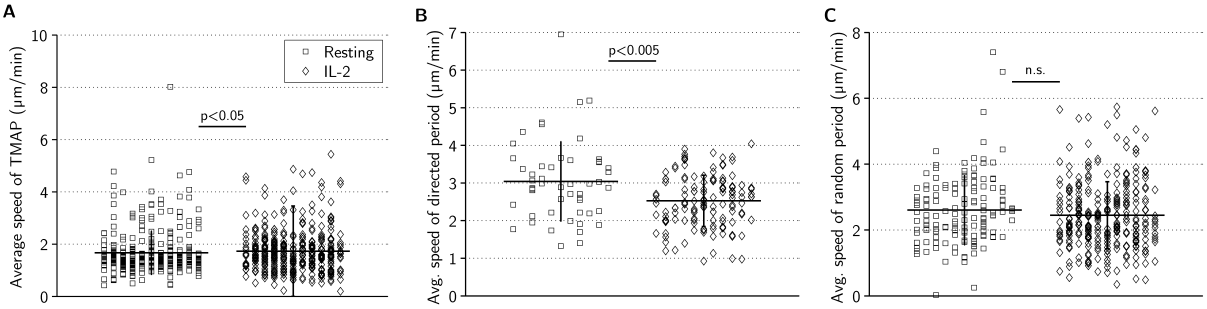

Figure S1. NK cell speed within different modes of migration. (A) Average speed within TMAPs for resting (squares, left) and IL-2-activated (diamonds, right) NK cells. **(B)** Average speed in directed migration periods for resting (squares, left) and IL-2-activated (diamonds, right) NK cells. **(C)** Average NK cell speed in random movement periods for resting (squares, left) and IL-2-activated (diamonds, right) NK cells. Resting NK cells had, on average, significantly faster migration speed in directed migration periods (3.0 μm/min for resting vs 2.5 μm/min for activated) compared to IL-2-activated NK cells. Significant differences were also observed in the average NK cell speed within TMAPs (1.7 μm/min for resting vs 1.7 μm/min for activated). The differences in migration speed of random movement periods (2.6 μm/min for resting vs 2.4 μm/min for activated) was not statistically significant. Horizontal and vertical bars represent mean and standard deviation.

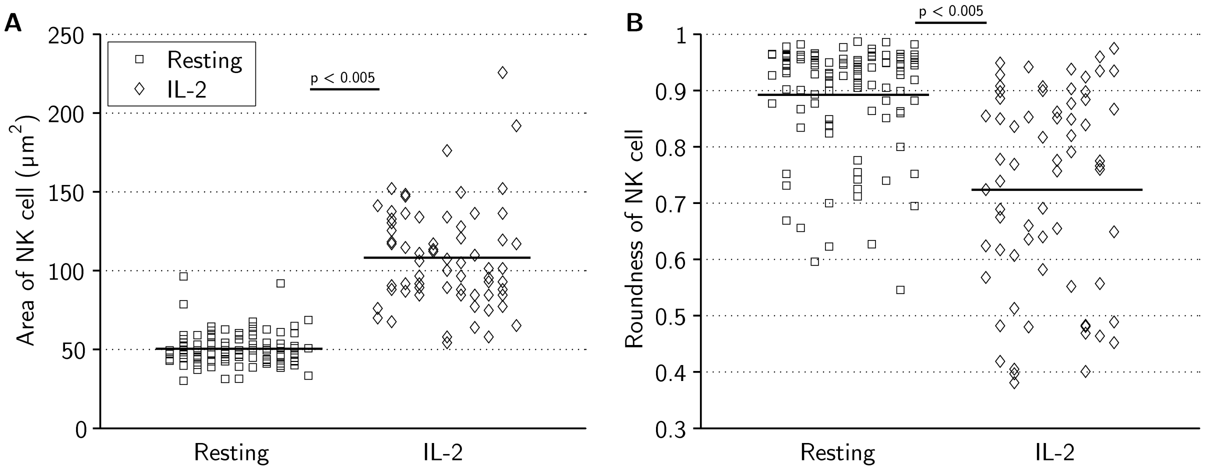

Figure S2. Resting NK cells are smaller and rounder than IL-2-activated NK cells. (A) NK cell area for resting (squares, left) and IL-2-activated (diamonds, right) NK cells. Horizontal bars represent mean values (50.5 μm^2 for resting and 108 μm^2 for activated NK cells, respectively). **(B)** NK cell roundness for resting (squares, left) and IL-2-activated (diamonds, right) NK cells. Horizontal bars represent mean values (0.89 for resting and 0.72 for activated NK cells, respectively). Regions of interest were manually drawn (in ImageJ) around each NK cell identified in the first frame of time-lapse movies from resting (n=106 NK cells from 8 wells) and activated (n=68 NK cells from 4 wells) NK cell area and roundness was measured. Roundness is defined as $4\pi A/P^2$, where A is the NK cell area and P is the NK cell perimeter, giving a perfect circular shape a roundness equal to 1.

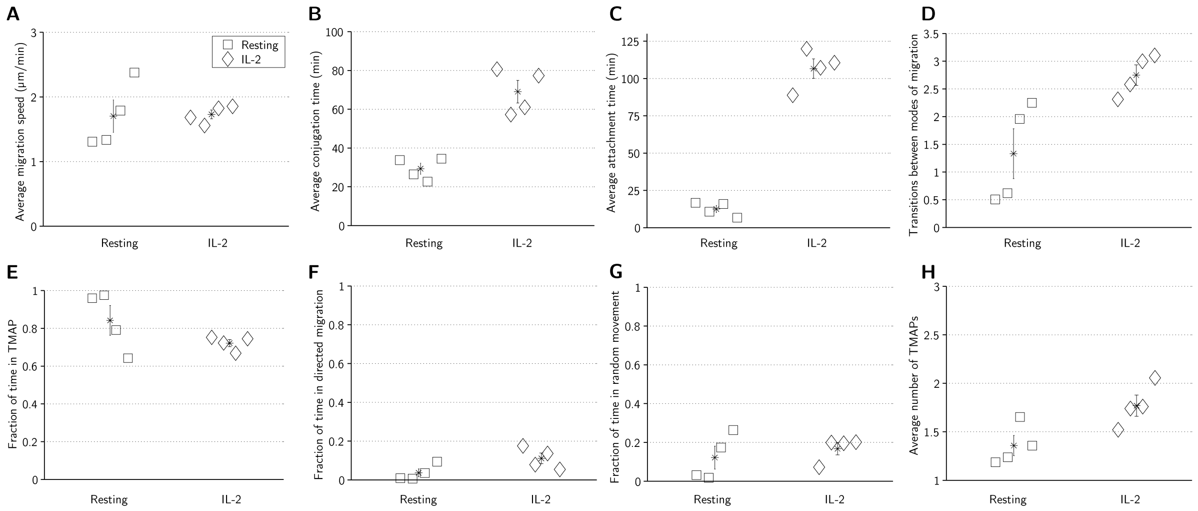

Figure S3. NK cell contact and migration dynamics plotted for different donors. (A) Average migration speeds for NK cells from donors in resting (*n*=4; squares, left) and IL-2-activated (*n*=4; diamonds, right) conditions. **(B)** Average duration of conjugation period for NK cells from donors in resting (squares, left) and IL-2-activated (diamonds, right) conditions. Similarly, the measures are broken down by donor and activation condition for **(C)**, average duration of attachment period, **(D)**, the average number of transitions between modes of migration, **(E)**, fraction of time spent in TMAP, **(F)**, fraction of time spent in directed migration, **(G)**, fraction of time in random movement, and **(H)**, average number of TMAPs per NK cell. Mean values are presented with standard error (mean±SEM).

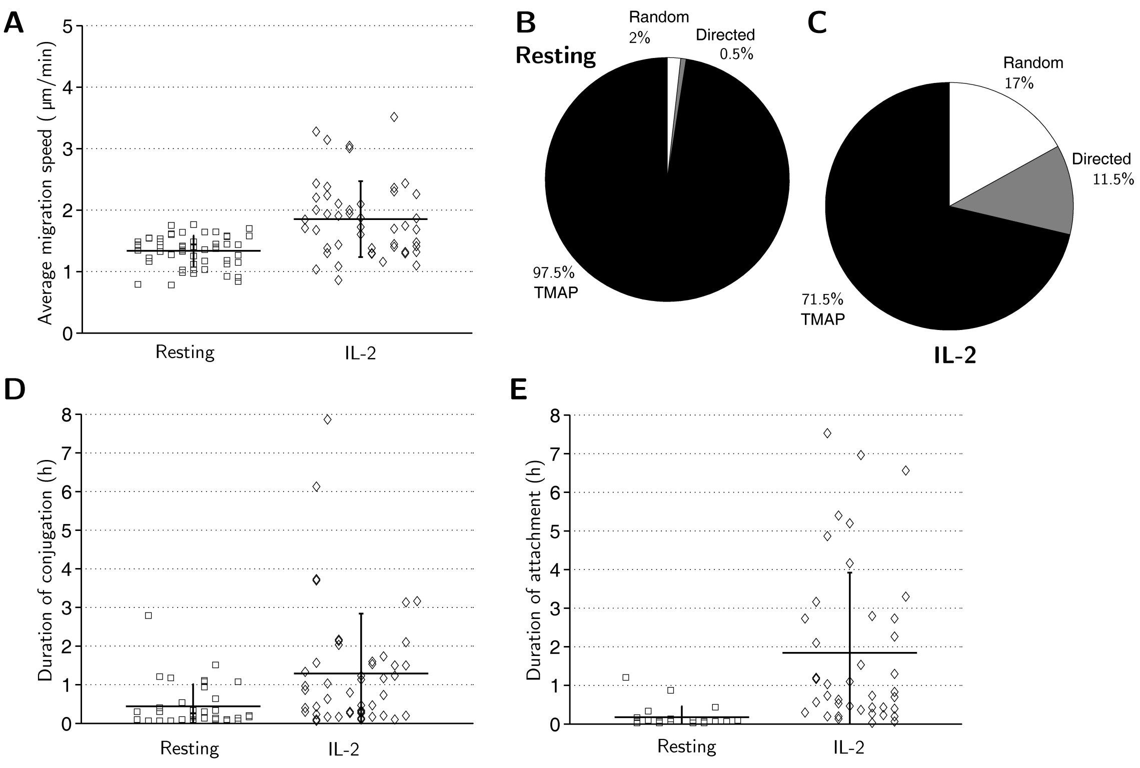

Figure S4. NK cells from the same donor change migration and contact dynamics after exposure to IL-2. (A) Average migration speed of NK cells obtained from the same donor in resting (squares, left) and IL-2-activated (diamonds, right) conditions. **(B, C)** Pie charts of the distribution of modes of migration for resting **(B)** and IL-2-activated **(C)** NK cells from the same donor. **(D)** Conjugation times for NK–target interactions made by resting (squares, left) and IL-2-activated (diamonds, right) cells. **(E)** Post-conjugation target cell attachment times for contacts made by resting (squares, left) and IL-2-activated (diamonds, right) NK cells.

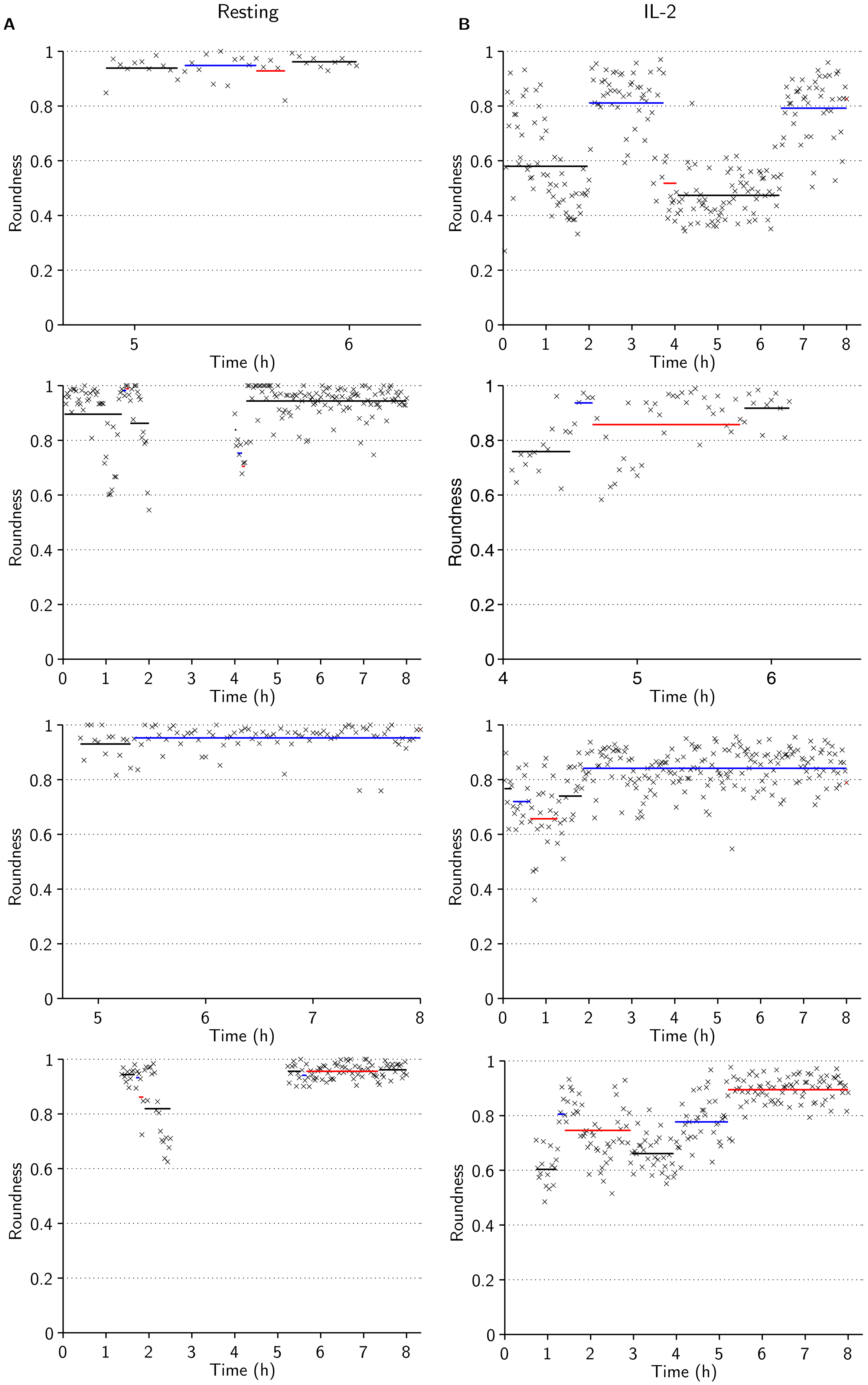
A
Resting
B
IL-2
Roundness
Time (h)
0
0.2
0.4
0.6
0.8
1
0 1 2 3 4 5 6 7 8

Figure S5. IL-2-activated NK cells become more round when conjugated to a target cell, while resting NK cells maintain their morphology throughout free migration and contact. (A, B) Plots showing roundness versus time for 4 randomly selected **(A)** resting and **(B)** IL-2-activated NK cells. The measured roundness is marked by x. Mean roundness values are plotted for conjugation periods (blue lines), attachment periods (red lines), and free migration (black lines).

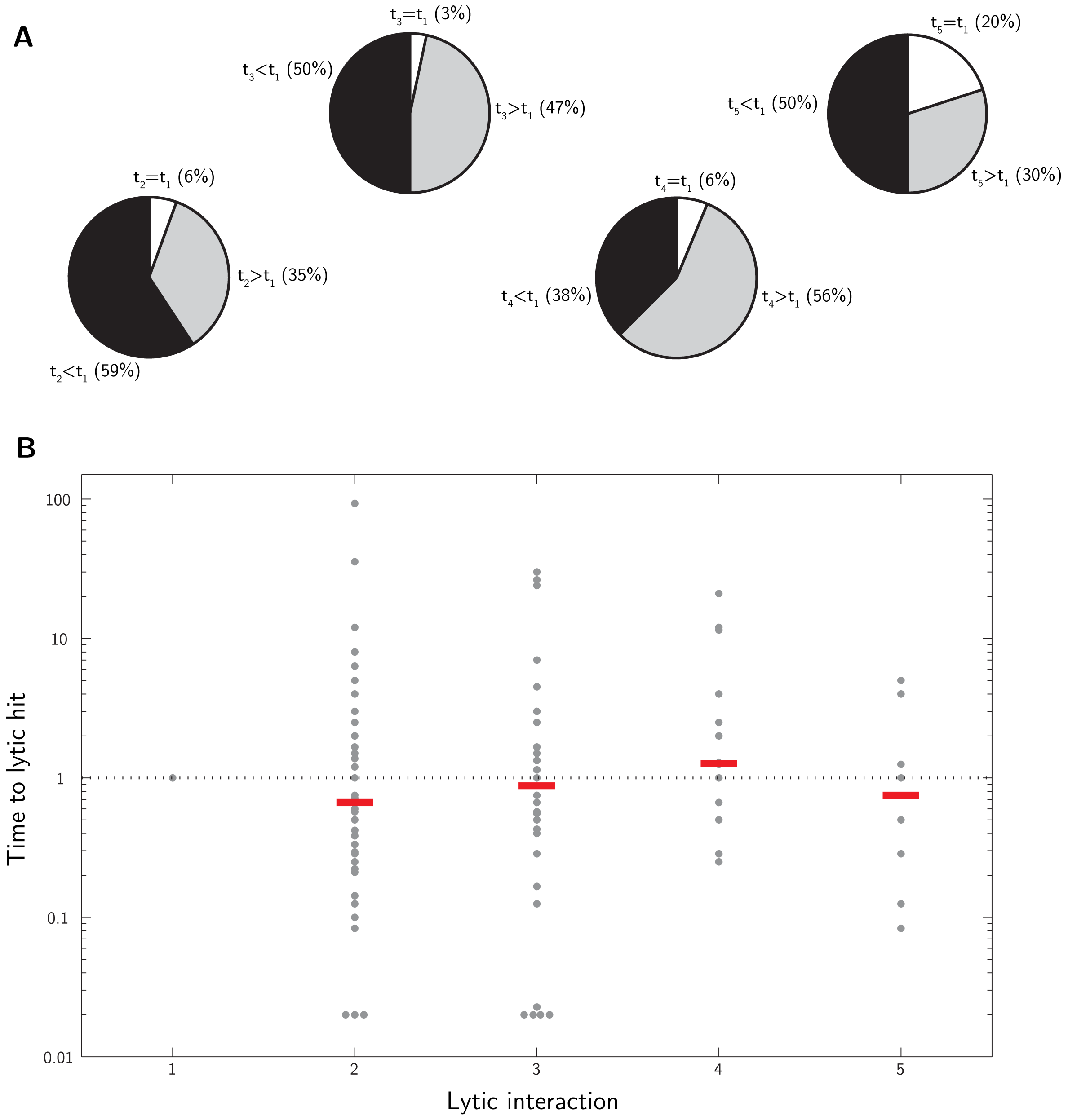

Figure S6. The majority of NK cells take more time to deliver the first hit. The times to lytic hit were compared for activated NK cells killing more than two targets. Only NK cells that killed the first target encountered and then at least one more target cells in direct sequence were considered. The timing between first and subsequent encounters was not considered. The time to lytic hit is the time from initiation of a contact to delivery of the lytic hit as measured by a distinct drop in target cell fluorescence (Vanherberghen, 2013, Blood). **(A)** Pie charts showing the percentage of cells with decreased (black sector), increased (grey sector) or equal (white sector) time to lytic hit compared to the first hit recorded. A majority of cells show decreased time for the second (n=54), third (n=30) and fifth (n=10) hit, but increased for the fourth hit (n=16). **(B)** Relative time to lytic hit for individual NK cells. The times for hits 2-5 have been normalized to the first time to lytic hit recorded for the individual NK cell. Red bars indicate median. In some occasions killing was faster than the time resolution of the experiment (2 min) and therefore set to 0 min. To be able to display also these point on a log scale these values where set to 0.02 (slightly below the shortest time >0 recorded).
